# Perpetrators using technology to sexually exploit children and animals: an emerging form of sexual abuse

**DOI:** 10.3389/fvets.2023.1285463

**Published:** 2023-12-18

**Authors:** Genevieve Bloxsom, Gemma McKibbin

**Affiliations:** Department of Social Work, University of Melbourne, Melbourne, VIC, Australia

**Keywords:** animal sexual abuse, child sexual abuse, child sexual exploitation, online sextortion, perpetrators use of technology

## Introduction

Veterinary medical professionals are in an ideal position to identify cases of animal sexual abuse. Despite this, a recent survey of 88 veterinary medical professionals indicated that most participants (81.6%) viewed animal sexual abuse knowledge as important, but the majority (88.1%) believed they did not have adequate information on the topic (1). This paper identifies a new form of animal sexual abuse that occurs in conjunction with child sexual abuse. Traditionally, perpetrators of child and animal sexual abuse have been identified as family members (2, 3) or people involved in “ritual abuse-torture” within family and community settings (4). That is, child and animal sexual abuse has previously been constructed as involving perpetrators who are known to the victim children and as occurring in face-to-face settings. This article describes a new form of child and animal sexual abuse which involves perpetrators who are unknown to the victims using technology to coerce children (up to the age of 18 years) into sexual activity with animals. The aim of this commentary paper is to describe this new form of abuse so that veterinary medical professionals can identify instances and assist the disruption of child and animal sexual abuse, including holding adult perpetrators to account.

## New form of child and animal sexual abuse

Levine's (5) report *Increasing the Efficacy of Investigations of Online Child Sexual Exploitation* sets out in detail the perpetrator methodology involved in this new form of technology-facilitated child and animal sexual abuse. Levine (5) explored federal child sexual abuse prosecution cases from a dataset published by the United States Sentencing Commission and conducted a qualitative analysis to characterize 19,830 successful prosecutions for child sexual abuse and exploitation crimes prosecuted between 2012 and 2019. In addition, the author analyzed more than 200 cases to identify potential investigative factors present in online child exploitation cases. The author also reviewed court documents to detail the operations of six different organized crime networks involved in sexual exploitation. The results of this analysis indicate that perpetrators use social media sites such as “MyLol” and YouTube to identify and communicate with vulnerable children. Adult perpetrators then provide children with links to unmoderated chat sites where the perpetrator portrays themselves to be under the age of 18. Children are manipulated to turn their webcams on, with the belief they are in a private conversation with a peer. Perpetrators then share previously recorded videos of children undressing and engaging in sexual activity whilst sending messages that match what is happening in the video, cementing the illusion that the perpetrators are other children livestreaming. For example, a 10-year-old receives a link to a chat site which they join and start sending instant messages to a person they think is their age. They are shown a video of a person their age undressing and think it is a livestream, and do not realize the instant messages are with an adult perpetrator and the video they are watching is a recording of a previous victim.

Next, these perpetrators request the victim child to undress and engage in sexual activity. The perpetrator uses images of this sexual activity to further exploit the child, including coercing them with threats to engage sexually with their household pets. Threats include saying that they will send video recordings of the victim performing sexual activity to their family and community, or that they will physically find and hurt the victim and their family. One perpetrator told a child that if she discontinued masturbating while livestreaming for him, he would tell everyone the victim was being sexually abused by her father and would send out videos he had made of her engaged in sexual activity with her pet dog. The sadistic, strategic and intentional abusive actions by these perpetrators are a form of sextortion.

## Case examples

Sextortion has been the focus of several recent media reports. In Australia, an adult male perpetrator used the internet to pose as a teenager to gather sexual content from children. This man then threatened to publish the content if the victim children did not follow his demands which included forcing a child to engage sexually with a dog (6). In England, a perpetrator used the app Snapchat to identify victim children. This man posed as a child to coerce other children to send sexually explicit photos, and used threats to release the content to force victim children to perform sexual acts on dogs (7). Another man in England used the apps Kik and Snapchat to force a child living in the United States to carry out a sexual act on a dog (8).

Some perpetrators have also made attempts to normalize children's sexual engagement with animals as part of the grooming process. In England, Sheehan (9) undertook a study involving semi-structured interviews with 22 male perpetrators who had been convicted of producing indecent images of children. The participants were identified by professionals working within the field and the data collected was thematically analyzed. One participant in Sheehan's (9) study is referred to as “Blair”, who had online chat logs with children which showed he had made victims engage in sexual activity on webcam with their dog. Blair would repeatedly send a child he met over the internet pictures of bestiality and links to websites dedicated to this. Blair later explained in an interview: “*I sent her images which I thought would sort of encourage, if you like, you're not alone, here's images of other children you know. I think that's what was going through my mind. Here's some images of children who have done it. It's not just you, other children do it”* [(9), p. 193]. This example demonstrates how perpetrators use child and animal sexual abuse imagery to groom victims.

## Trends indicating increasing prevalence

The Canadian Center for Child Protection (10) categorizes the severity of child sexual exploitation material into four groups with the highest being “Severity 4”, which involves extreme sexual assaults, as well as bestiality, bondage, weapons and defecation. In their 2009 report, 2.7% (*n* = 111) of images (*n* = 4,110) were “Severity 4”. Eight years later, their 2016 report recorded 2.23% (*n* = 974) of images and videos (*n* = 43,760) as “Severity 4”. Whilst it remains unknown what portion of these “Severity 4” videos and images included technology-facilitated child and animal abuse, these data indicate that prevalence is likely increasing over time. As more of these cases emerge it is vital that professionals responsible for responding to this abuse can clearly identify the adult perpetrator as the person responsible for the harm, rather than blaming victim children who are made to sexually engage with animals. The accurate identification of the adult perpetrator is important to avoid victim blaming, and to assist law enforcement efforts to disrupt the sexual exploitation and hold adult perpetrators accountable.

## Need for training and advice on terminology

Veterinary medical professionals may treat an animal where suspicion of child and animal sexual abuse arises and could struggle with correct identification of the abuse and experience a lack of guidance on appropriate language. Despite other medical professionals such as pediatricians receiving training to enhance their capacity to respond to child sexual abuse (11), veterinarians receive insufficient professional development opportunities relating to child and animal sexual abuse (1). We offer some advice about the language vets can use to describe this form of abuse.

The language used to describe this emerging form of child and animal sexual abuse is significant because sexual abuse and exploitation terminology impacts how a problem is conceptualized, influencing what responses are considered appropriate (12). We argue for the adoption of the Terminology Guidelines developed by the Interagency Working Group (13). These guidelines were developed by leading global organizations—like the United Nations Committee on the Rights of the Child and the International Center for Missing and Exploited Children—which operate in the field of child sexual abuse and exploitation. The guidelines are based on the critical analysis of child sexual abuse and exploitation terminology and have informed how the words “victim, survivor and perpetrator” are used. The guidelines do not provide advice on all terminology associated with the new form of child and animal sexual abuse described in this paper. In these instances, we provide advice about appropriate language when documenting this form of abuse.

It is recommended that the term “victim” is used when describing the child who was made to sexually engage with an animal. It may also be appropriate to use the term “survivor” if that individual child feels the word correctly reflects their experience, however this term should not be automatically applied to every situation. For some children, the abuse may be continuing or the child may still be experiencing harmful impacts of the abuse (such as self-harm, attempts to end their life, social isolation, drug and alcohol use and further victimization) and constructing them as a “survivor” may diminish the extent of the impact or continued abuse. It is also understood that animals share this “victim” or “survivor” position with children.

The words “forced” and “manipulated” have been used throughout this article to represent the coercive nature of this form of abuse and to keep perpetrators' actions visible. The term “sexual abuse” has only been used to describe perpetrator actions. When describing the acts that the child was made to do, the term “sexually engage with animals” has been adopted to avoid constructing the victim as a person who sexually abuses animals, as this view would further stigmatize the child and contribute to victim-blaming. Given the extreme power imbalances present in these cases and the complete lack of agency victim children have, the terms “bestiality” and “perpetrator” should only be used to describe the behavior and identity of adults who force children to sexually engage with animals.

It is crucial that veterinary medical professionals are considerate of the language used to describe child and animal sexual abuse, especially when communicating their concerns and assessment to families, colleagues, child protection departments and police. How language is used should consistently be respectful and uphold the dignity of the child. With deliberate choice of language, child and animal sexual abuse can be accurately recorded by veterinary medical professionals and appropriately responded to within a multidisciplinary setting.

## Conclusion

This article has presented an emerging form of child and animal sexual abuse. We have identified how perpetrators target children via everyday social media platforms and coerce them to video themselves sexually engaging with their household pets. Case studies have been provided which demonstrate the methods that perpetrators use, the globalization of the issue and how it appears that prevalence rates are increasing. Advice about preferred terminology to describe the abuse is discussed in relation to reducing the risk of child victim blaming and keeping perpetrators' abusive actions visible. This type of child and animal sexual abuse warrants further research to generate knowledge about early identification and the assistance that children and animals require to support their healing process. Further, the role of multiagency work involving veterinary medical professionals, police and child protection to achieve the best outcomes for victim children and animals could be explored.

## Author contributions

GB: Conceptualization, Project administration, Visualization, Writing – original draft. GM: Conceptualization, Project administration, Supervision, Visualization, Writing – review & editing.
